# Tie Rod-Equivalent Non-Linear Constitutive Law for Uniformly Loaded Cables

**DOI:** 10.3390/ma14195502

**Published:** 2021-09-23

**Authors:** Pietro Croce

**Affiliations:** Department of Civil and Industrial Engineering, University of Pisa, Largo Lazzarino, 1-56122 Pisa, Italy; p.croce@ing.unipi.it; Tel.: +39-335-5345611

**Keywords:** cable, equivalent stiffness, Dischinger’s modulus, nonlinear behavior, virtual work principle, parabolic cable, overhead lines, suspension bridge, Irvine formula

## Abstract

Cables are typically used in engineering applications as tensile members. Relevant examples are the main cables of suspension bridges, the stays of cable-stayed bridges, the load-bearing and stabilizing cables of tensile structures, the anchor cables of floating mooring structures, the guy-ropes for ship masts, towers, and wind turbines, the copper cables of electrical power lines. Since cables are characterized by non-linear behavior, analysis of cable structures often requires advanced techniques, like non-linear FEM, able to consider geometric non-linearity. Nevertheless, a traditional simplified approach consists in replacing the cable with an equivalent tie rod, characterized by a suitable non-linear constitutive law. Currently used equivalent constitutive laws have been derived by Dischinger, Ernst and Irvine. Since the equivalence is restricted to taut cables, characterized by small sag to chord ratios, these traditional formulae are not appropriate for uniformly loaded sagging cables: the main cables of suspension bridges are a particularly emblematic case. Despite some recent attempts to find more refined solutions, the problem is still open, since closed form solutions of general validity are not available. In the paper, general analytical formulae of the non-linear constitutive law of the equivalent tie rod are proposed, distinguishing two relevant cases, according as the length of the cable can vary or not. The expressions, derived by applying the general form of the theorem of virtual work, can be applied independently on the material, on the sag to chord ratio, on the load intensity and on the stress level, so allowing the replacement of the whole cable with a single equivalent tie rod. The expressions are critically discussed referring to a wide parametric study also in comparison with the existing formulae, stressing the influence of the most relevant parameters.

## 1. Introduction

Cables are widely used in engineering fields as typical load bearing tensile members: relevant examples are the main cables of suspension bridges, the stays of cable-stayed bridges, the load-bearing and stabilizing cables of tensile structures, the anchor cables of floating mooring structures, the guy-ropes for masts, ship masts, towers, and wind turbines, the copper cables of electrical power lines, and so on. Nowadays, depending on the application, cables can be made resorting to a large variety of different materials. Suitable materials span from the historical ones, like natural fibers (cotton, flax, jute, silk), the use of which is lost in mists of time, to the modern ones, like high-strength steel, up to the most advanced ones, like aramid, glass, polyester, and carbon fiber. Innovative structural materials are more and more proposed: for suspension and cable-stayed bridges [1,2], for other general structural applications [3,4], as well as for strengthening, restoration and repair of historical and heritage buildings [5], even considering hybrid solutions [6].

Since cables are characterized by geometric non-linear behavior, analysis of cable structures requires suitable advanced techniques, like non-linear FEM, able to consider this kind of non-linearity, while the material is assumed linear elastic. A traditional and very effective approach to simplify the structural model consists in replacing the cable, whose behavior is governed by geometric nonlinearity, with an equivalent tie rod connecting the ends of the cable, characterized by a suitable non-linear constitutive law. The rationale of this approach is emulating the apparent along the chord stiffness of the cable with by the axial stiffness of the equivalent tie rod: it is crystal clear that in this way, the complexity of structural models can be strongly reduced, as it occurs in finite element analyses, where geometric non-linearities can be captured by representing the cable with a unique equivalent element characterized by material non-linearity.

Under the basic assumptions that bending and shear stiffnesses of the cable are negligible in comparison with its axial stiffness, and that cables’ configurations are characterized by small sag to chord ratios, the assessment of the non-linear constitutive law of the equivalent tie rod has been the subject of several studies [7,8,9,10], which provide suitable approximate expressions for the tangent or the secant elastic modulus of the equivalent straight tie. Widely used classical expressions have been derived by Dischinger [7,8] and Ernst [9], considering a horizontal cable. Said a the chord length, $A_{0}$ the area of the cross section, and p the unit weight of the cable, acting in the vertical direction, Dischinger [7,8] expressed the equivalent elastic tangent modulus, $E_{t,eq}$, as ratio between the variation, $d\sigma_{0}$, of the horizontal component of the normal stress in the cable, $\sigma_{0}$, and the variation of the deformation, dε, along the chord:

(1) $$E_{t,eq}\left(\sigma_{0}\right)=\frac{d\sigma_{0}}{d\varepsilon }=\frac{E}{1+\frac{{\left(p\;a\right)}^{2}}{12\;A_{0}^{2}\;\sigma_{0}^{3}}\;E},$$

being E is the elastic modulus of the cable material. Of course, from the equilibrium equations it derives that $\sigma_{0}$ is independent on the considered cross section. Considering a variation of the normal stress between $\sigma_{0i}$ and $\sigma_{0f}$, Ernst [9], following the Dischinger’s approach, derived the equivalent secant modulus as:

(2) $$E_{s,eq}=\frac{\Delta \sigma_{0}}{\Delta \varepsilon }=\frac{E}{1+\frac{{\left(p\;a\right)}^{2}}{24\;A_{0}^{2}\;\sigma_{0i}^{3}}\;\frac{1+\bar{\sigma }}{{\bar{\sigma }}^{2}}E},$$

where

(3) $$\bar{\sigma }=\frac{\sigma_{0f}}{\sigma_{0i}}.$$

**Figure 1:**
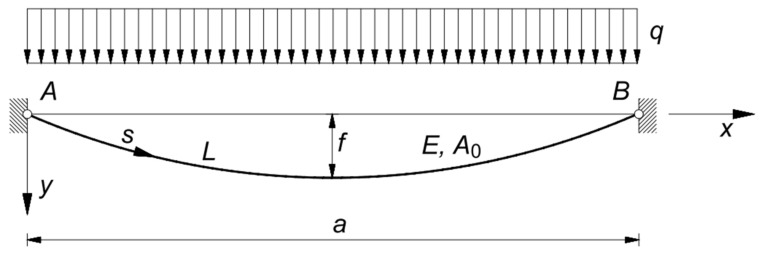
Deformed (parabolic) configuration of a cable subject to a uniformly distributed load q.

**Figure 2:**
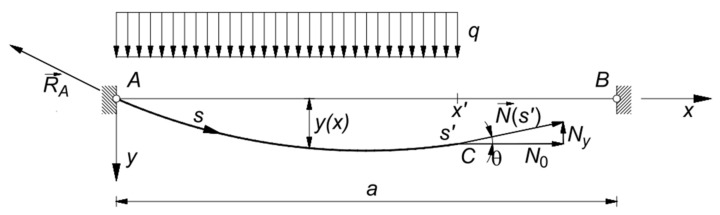
System of forces acting on the cable portion AC.

As these studies are based on simplified assumptions, the equivalence is strictly restricted to stretched cables subjected to the self-weight, so characterized by catenary shape, even if Equations (1) and (2) have been often adopted also for uniformly loaded cables, in case the sag to chord ratio is small enough to approximate the actual deformed shape, which is a parabola, with a catenary. In fact, let be the uniformly distributed load q acting perpendicularly to the chord AB of the cable, and assume the axes, x and y, of a 2D Cartesian coordinate system, whose origin is in A, given by AB, and q, respectively, the configuration $y\left(x\right)$ of the cable under q is given by the parabolic funicular curve (Figure 1).

(4) $$y\left(x\right)=\frac{q\;}{2\;N_{0}}x\left(a-x\right)=4\;\frac{f\;}{a}x\left(1-\frac{x}{a}\right),$$

where f is the sag of the cable and $N_{0}$,

(5) $$N_{0}=\frac{q\;a^{2}\;}{8\;f},$$

is the x-component of the normal force N in the cable, which is obtained from the equilibrium condition of the portion AC of the cable (Figure 2). For the sake of simplicity, it can be assumed that x is horizontal, and y is vertical.

In principle, Equation (4) is derived assuming the cable inextensible, i.e., having infinite axial rigidity, but it can be properly modified to take into account the Hooke’s law for the cable [10], so making unnecessary the inextensibility hypothesis. Equation (4) thus applies independently on the cable material properties the material is elastic, and the initial length of the cable, L, is greater than the chord, a: L>a, provided it is written referring to the final, stretched, configuration. The condition L>a ensures that if the external load is nil, the cable is unstressed. In case L<a, since the installation of the cable is conditional on suitable pre-strain, ${\bar{\varepsilon }}_{p}$, of the cable itself

(6) $${\bar{\varepsilon }}_{p}=\frac{a}{L}-1,$$

elastic deformations need must be explicitly considered.

To improve the equivalent model, widening its field of application, Irvine [10] suggested to express the tangent elastic modulus in the form

(7) $$E_{t,eq1}=\frac{d\sigma }{d\varepsilon }=\frac{E}{1+\frac{\lambda^{2}}{12}},$$

where the deformed configuration of the cable is considered by means of the so-called characteristic parameter of the cable, $\lambda^{2}$

(8) $$\lambda^{2}={\left(\frac{q\;a}{N_{0}}\right)}^{2}\frac{a\;E}{\sigma_{0}\;L_{e}},$$

where $L_{e}$ is the virtual length of the cable, depending on the arc length generalized coordinate s,

(9) $$L_{e}=\overset{a}{\underset{0}{\int }}{\left(\frac{ds}{dx}\right)}^{3}dx.$$

Recalling Equation (4), after some elementary manipulations, Equation (8) gives

(10) $$L_{e}=\overset{a}{\underset{0}{\int }}{\left(1+y{’}^{2}\right)}^{\frac{3}{2}}dx=\frac{a}{8}\;\left\{\sqrt{1+{\left(4\;\frac{f}{a}\right)}^{2}}\;\left[5+2\;{\left(4\;\frac{f}{a}\right)}^{2}\right]+\frac{3}{4}\;\frac{a}{f}\;\mathrm{asinh}\left(4\;\frac{f}{a}\right)\right\},$$

which, in case a≫f, can be satisfactorily approximated, following Irvine, by

(11) $$L_{e}≅a\;\left[1+8\;{\left(\frac{f}{a}\right)}^{2}\right].$$

For the sake of completeness, we recall that actual length of the cable L is

(12) $$L=\overset{a}{\underset{0}{\int }}{\left(1+y{’}^{2}\right)}^{\frac{1}{2}}dx=\frac{a}{2}\;\left[\sqrt{1+{\left(4\;\frac{f}{a}\right)}^{2}}+\frac{1}{4}\;\frac{a}{f}\;\mathrm{asinh}\left(4\;\frac{f}{a}\right)\right],$$

which can be estimated, when a>0.1 f, by

(13) $$L≅a\;\left[1+\frac{8}{3}\;{\left(\frac{f}{a}\right)}^{2}\right],$$

When applicable, Irvine expression, Equation (7), is generally more reliable than the Dischinger formula, Equation (1); anyhow, not fully considering the influence of the modifications of the cable configurations on the position of the external loads, these classical expressions are both dependent on important limitations, which limit their field of application to taut cables. It must be remarked that relevant structures can be characterized by uniformly loaded sagging cables, where the cited approaches are not appropriate: the main cables of suspension bridges are particularly emblematic cases. For this reason, several theoretical and numerical studies have been carried out [11,12,13,14,15,16,17,18,19,20,21], with the aim of achieving more general solutions, valid also for relaxed cables. Moreover, in the last years, further improvements of the classical expressions have been proposed, in view of the implementation of appropriate non-linear cable elements in finite element analysis [22,23,24,25], also discussing the influence of bending stiffness and shear stiffness [26,27]. Anyhow, when significant, the effects of bending stiffness and shear stiffness on the cable configuration can be inferred as local perturbations of the solution derived disregarding them.

It must be remarked that uniformly loaded sagging cables characterize relevant structures, where the cited approaches are not appropriate: the main cables of suspension bridges are particularly emblematic cases. Despite the already cited relevant literature [22,23,24,25] illustrating some attempts to find more refined solutions, the problem is still open, since closed form solutions of general validity are not yet available.

In the paper, a general analytical expression of the non-linear constitutive law of an equivalent tie rod simulating uniformly loaded cables is proposed, distinguishing two relevant cases, according to whether the length of the cable can vary or not:

- the first case, which is rather new, corresponds to the case when an extremity of the cable is fixed and the final section on the other end can move over a fixed pulley, so that the chord length cannot vary;
- the second case, which is the one traditionally considered, corresponds to the case when the cable length is fixed.

The expressions, derived by applying the general form of the theorem of virtual work, can be applied to uniformly loaded cables, independently on the material, on the sag to chord ratio, and on the load intensity, so allowing the replacement of the whole cable with a single equivalent tie rod. The theoretical approach is a general implementation of a procedure already successfully proposed to derive the equivalent along the chord stiffness of horizontal [28] and inclined stays [29], loaded by the self-weight.

It must be remarked that the proposed approach can be also a powerful tool to obtain reliable prior information, in view of the multifaceted implementation of advanced Bayesian updating techniques for the identification of mechanical parameters [30,31,32,33,34].

The obtained expression is thus critically discussed in some relevant case studies, also in comparison with the existing formulae, as well as with the results of non-linear finite element analysis, stressing the influence of the most relevant parameters as well as its advantages.

## 2. The Non-Linear Constitutive Laws of the Equivalent Tie-Rod

The non-Linear constitutive law of the equivalent tie-rod can be derived by applying the theorem of virtual works to the cable, considering a horizontal virtual relative displacement between the cable ends, dx [28,29]. Without loss of generality, we assume the end A is fixed, so that the relative displacement is at the end B, $dx_{B}$.

**Figure 3:**
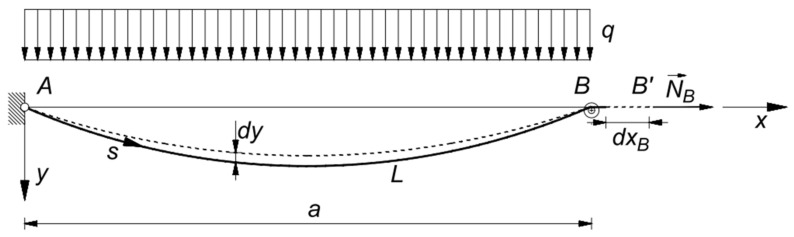
Virtual deformations of a cable running on a fixed pulley in B: da=0.

**Figure 4:**
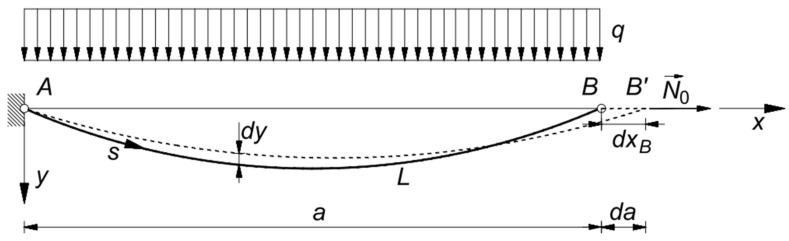
Virtual deformations of a cable with ends fixed in A and B.

As anticipated, two relevant cases can be envisaged, depending on the boundary conditions in B, according as whether the distance between A and B, i.e., the chord length a, is fixed or not:

(a) if the chord length is fixed, and the cable runs on a fixed pulley in B, the sole effect of the virtual displacement $dx_{B}$ is a variation of the cable length L: $dL=dx_{B}$, and da=0 (Figure 3);
(b) if cable ends, A and B, are fixed, the effect of the relative displacement is a variation of the cable chord a: $da=dx_{B}$ (Figure 4).

The former case typically describes the behavior of cables during the tightening phase, the latter the behavior of structural cables in usual operational conditions.

### 2.1. Theorem of Virtual Work for the Cable

Referring to both previously mentioned cases, the virtual work equation for the cable can be expressed in the form,

(14) $$\left({\vec{N}}_{B}+d{\vec{N}}_{B}\right)\cdot d{\vec{x}}_{B}+\overset{a}{\underset{0}{\int }}\vec{q}\cdot d\vec{y}\;dx=\overset{}{\underset{V}{\int }}\sigma d\varepsilon \;dV,$$

where $d\vec{y}$ is the variation of the cable ordinate due the modification of the cable configuration, σ is the normal stress, dε the variation of the longitudinal strain, and V the volume. Evidently, if the cable is assumed inextensible, dε=0, and the virtual work of normal stresses is nil.

Since $\vec{q}$ and $\vec{y}$ are parallel and $d{\vec{N}}_{B}\cdot d{\vec{x}}_{B}$ can be disregarded in comparison with ${\vec{N}}_{B}\cdot d{\vec{x}}_{B}$, Equation (14) becomes

(15) $${\vec{N}}_{B}\cdot d{\vec{x}}_{B}+\overset{a}{\underset{0}{\int }}q\;dydx=\overset{}{\underset{V}{\int }}\sigma d\varepsilon \;dV.$$

The virtual displacement $dx_{B}$ in B causes a variation of the cable configuration, dy, which is the effect of the variations of the chord length, da, as well as of the normal force, dN. The effect of the previously occurring elastic deformations is implicitly considered, setting the length of the “inextensible” cable equal to its final, deformed, length.

The total variation of the ordinate dy of a point of the cable, whose abscissa is x, depends on the variation of the chord length da, and on the variation of the horizontal component of the normal force $dN_{0}$. From another perspective, the total variation of the ordinate dy can be seen as sum of two contributions, the one associated with the variation of the configuration of the cable, assumed inextensible, $dy_{in}$, the other associated with the elastic stretching of the cable, $dy_{el}$. It must be remarked that, while the variation of the horizontal component of the normal force, $dN_{0}$, influences both the inelastic contribution, $dy_{in}$, and the elastic contribution, $dy_{el}$, the variation of the chord length, da, affects only $dy_{in}$, being negligible its effect on $dy_{el}$.

The contribution due to the modification of the inextensible cable configuration, can be obtained differentiating the cable equation (Equation (4)):

(16) $$dy_{in}=\frac{\partial y}{\partial a}da+\frac{\partial y}{\partial N_{0}}dN_{0},$$

which reduces to

(17) $$dy_{in}=\frac{\partial y}{\partial N_{0}}dN_{0},$$

in the previously cited case (a), when the chord length is fixed: da=0.

The variation of the cable ordinate, $dy_{el}$, due to the elastic deformation is a function of the vertical component of the normal force only. A very good estimate of the contribution of the variation of the elastic deformations can be obtained approximating the parabola with a catenary, whose total weight is equal to total applied load. The unit self-weight of the equivalent catenary $q^{*}$ is thus

(18) $$q^{*}=p\frac{a}{L_{c}},$$

being $L_{c}$ is the length of the catenary,

(19) $$L_{c}=2\;\frac{N_{0}}{p^{*}}\;\sinh\left(\frac{q^{*}\;a}{2\;N_{0}}\right),$$

from which,

(20) $$q^{*}=2\;\frac{N_{0}}{a}\;\mathrm{arcsinh}\left(\frac{q\;a}{2\;N_{0}}\right),$$

which is independent on the Lagrangian coordinate s.

As suggested by Irvine [10], $y_{el}\left(s\right)$ can be expressed as

(21) $$\Delta y_{el}\left(s\right)=\frac{N_{0}}{E\;A_{0}}y^{\prime }\left(0\right)s-q^{*}\frac{s^{2}}{2\;E\;A_{0}}.$$

Differentiating Equation (21) with respect to $N_{0}$ and disregarding the effects of the normal force variation on $pq^{*}$, we find

(22) $$dy_{el}=\left(\frac{y’\left(0\right)\;}{E\;A_{0}}+\frac{N_{0}}{E\;A_{0}}\frac{dy’\left(0\right)}{dN_{0}}\right)s\;dN_{0}.$$

Integrating on the cable length, we obtain

(23) $$\begin{array}{ll} \overset{a}{\underset{0}{\int }}q\frac{\partial y_{el}}{\partial N_{0}}dx≅ & \overset{L_{c}}{\underset{0}{\int }}q^{*}\frac{\partial y_{el}}{\partial N_{0}}ds\;dN_{0}=q^{*}\left(\frac{y’\left(0\right)\;}{E\;A_{0}}+\frac{N_{0}}{dN_{0}}\frac{dy’\left(0\right)}{E\;A_{0}}\right)\overset{L_{c}}{\underset{0}{\int }}s\;ds\\ & =\frac{q^{*}\;L_{c}^{2}}{2\;E\;A_{0}}\;\left(\sinh\left(\frac{q^{*}\;a}{2\;N_{0}}\right)-\frac{q^{*}\;a}{2\;N_{0}}\;\cosh\left(\frac{q^{*}\;a}{2\;N_{0}}\right)\right)=\frac{q^{*}\;L_{c}^{2}}{2\;E\;A_{0}}\;\left(\frac{q\;a}{2\;N_{0}}-\sqrt{1+{\left(\frac{q\;a}{2\;N_{0}}\right)}^{2}}\;\mathrm{arcsinh}\left(\frac{q\;a}{2\;N_{0}}\right)\right)\\ & =\frac{q^{2}\;a^{3}}{4\;N_{0}\;E\;A_{0}}\;\left(\frac{\frac{q\;a}{2\;N_{0}}}{\mathrm{arcsinh}\left(\frac{q\;a}{2\;N_{0}}\right)}-\sqrt{1+{\left(\frac{q\;a}{2\;N_{0}}\right)}^{2}}\;\right) \end{array}$$

Obviously, the present study duly considers effects of elastic deformations: in fact, in the following, only the variation of the integration limits associated with the elastic elongation of the cable are disregarded, according to common practice.

### 2.2. The Non-Linear Constitutive Law for a Cable Running on a Fixed Pulley

In case the cable runs on a fixed pulley at its end B (Figure 3), it results in da=0. Recalling Equations (4), (5), and (17), Equation (15) becomes:

(24) $$N_{B}\;dl+\overset{a}{\underset{0}{\int }}qdy_{el}\;dx+\overset{a}{\underset{0}{\int }}q\frac{\partial y}{\partial N_{0}}dN_{0}\;\;dx=\overset{L}{\underset{0}{\int }}\frac{N}{A_{0}}\frac{dN}{E\;A_{0}}A_{0}\;ds,$$

which reduces, after some elementary passages, to

(25) $$N_{0}\;\sqrt{1+y^{\prime }{\left(a\right)}^{2}}\;dx+\overset{a}{\underset{0}{\int }}q\frac{\partial y_{el}}{\partial N_{0}}dN_{0}\;\;dx+\overset{a}{\underset{0}{\int }}q\frac{\partial y}{\partial N_{0}}dN_{0}\;\;dx=\overset{L}{\underset{0}{\int }}\frac{N_{0}}{\cos\theta E\;A_{0}}\;\frac{dN_{0}}{\cos\theta }\;ds,.$$

where θ is the angle between the deformed configuration and the x-axis (Figure 2).

Further manipulations of Equation (25) lead to:

(26) $$N_{0}\;\sqrt{1+y^{\prime }{\left(a\right)}^{2}}\;dx+\overset{a}{\underset{0}{\int }}q\frac{\partial y_{el}}{\partial N_{0}}dN_{0}\;\;dx+\overset{a}{\underset{0}{\int }}q\frac{\partial y}{\partial N_{0}}dN_{0}\;\;dx=\frac{N_{0}\;dN_{0}}{E\;A_{0}}\overset{a}{\underset{0}{\int }}\;{\left(1+y{’}^{2}\right)}^{\frac{3}{2}}\;dx.$$

Evaluating separately the individual contributions of each relevant term of Equation (26), we obtain

(27) $$N_{0}\;\sqrt{1+y^{\prime }{\left(a\right)}^{2}}\;dx=N_{0}\;\sqrt{1+{\left(\frac{q\;a}{2\;N_{0}}\right)}^{2}}\;da,$$

(28) $$\overset{a}{\underset{0}{\int }}q\frac{\partial y}{\partial N_{0}}dN_{0}\;dx=\overset{a}{\underset{0}{\int }}\frac{q^{2}x}{2\;N_{0}^{2}}\left(x-a\right)\;dx\;dN_{0}=-\frac{q^{2}a^{3}}{12\;N_{0}^{2}}\;dN_{0},$$

and

(29) $$\overset{a}{\underset{0}{\int }}\;{\left(1+y{’}^{2}\right)}^{\frac{3}{2}}\;dx=\frac{\;1}{8}\;\left\{a\;\sqrt{1+{\left(\frac{q\;a}{2\;N_{0}}\right)}^{2}\;}\left[5+2{\left(\frac{q\;a}{2\;N_{0}}\right)}^{2}\right]+6\;\frac{N_{0}}{q}\;\mathrm{asinh}\left(\frac{q\;a}{2\;N_{0}}\right)\right\},$$

which obviously corresponds to Equation (10), and finally, recalling Equation (29):

(30) $$\begin{array}{ll} \sqrt{1+{\left(\frac{q\;a}{2\;N_{0}}\right)}^{2}}\;dx & \\ & =\frac{q^{2}a^{3}}{12\;N_{0}^{3}}\;dN_{0}\\ & +\frac{\;dN_{0}}{8\;E\;A_{0}}\;\{a\;\sqrt{1+{\left(\frac{q\;a}{2\;N_{0}}\right)}^{2}\;}\left[5+2{\left(\frac{q\;a}{2\;N_{0}}\right)}^{2}\right]+\;6\frac{N_{0}}{q}\;\mathrm{asinh}\left(\frac{q\;a}{2\;N_{0}}\right)\\ & -\frac{2\;q^{2}a^{3}}{N_{0}^{2}}\left(\frac{\frac{q\;a}{2\;N_{0}}}{\mathrm{asinh}\frac{q\;a}{2\;N_{0}}}-\sqrt{1+{\left(\frac{q\;a}{2\;N_{0}}\right)}^{2}\;}\right)\}. \end{array}$$

By dividing both members of Equation (30) by a, and recalling that

(31) $$d\varepsilon =\frac{dx}{a},$$

is the apparent along the chord deformation of the cable, and of the equivalent tie-rod, it results

(32) $$\begin{array}{ll} \sqrt{1+{\left(\frac{\varrho \;a}{2\;\sigma_{0}}\right)}^{2}}\;d\varepsilon & =\frac{\varrho^{2}a^{2}}{12\;\sigma_{0}^{3}}\;d\sigma_{0}\\ & +\frac{\;d\sigma_{0}}{8\;E\;}\;\{\;\sqrt{1+{\left(\frac{\varrho \;a}{2\;\sigma_{0}}\right)}^{2}\;}\left[5+2{\left(\frac{\varrho \;a}{2\;\sigma_{0}}\right)}^{2}\right]+6\;\frac{\sigma_{0}}{\rho \;a}\;\mathrm{asinh}\left(\frac{\varrho \;a}{2\;\sigma_{0}}\right)\\ & -\frac{2\;\varrho^{2}a^{2}}{\sigma_{0}^{2}}\left(\frac{\frac{\varrho \;a}{2\;\sigma_{0}}}{\mathrm{asinh}\left(\frac{\varrho \;a}{2\;\sigma_{0}}\right)}-\sqrt{1+{\left(\frac{\varrho \;a}{2\;\sigma_{0}}\right)}^{2}\;}\right)\}, \end{array}$$

where ϱ is the specific load, i.e., the ratio between the uniformly distributed load and the area of the cable

(33) $$\varrho =\frac{q}{A_{0}}.$$

The non-linear constitutive law of the equivalent tie-rod, expressed in terms of tangent elastic modulus, is thus:

(34) $$\begin{array}{l} E_{t,eq,0}=\frac{d\sigma_{0}}{d\varepsilon }\\ =\frac{\;\sqrt{1+{\left(\frac{\varrho \;a}{2\;\sigma_{0}}\right)}^{2}}\;E}{\frac{1}{3}{\left(\frac{\varrho \;a}{2\;\sigma_{0}}\right)}^{2}\frac{E}{\sigma_{0}}+\frac{1}{8}\;\sqrt{1+{\left(\frac{\varrho \;a}{2\;\sigma_{0}}\right)}^{2}\;}\left[5+2{\left(\frac{\varrho \;a}{2\;\sigma_{0}}\right)}^{2}\right]+\frac{3}{4}\;\frac{\sigma_{0}}{\rho \;a}\;\mathrm{asinh}\left(\frac{\varrho \;a}{2\;\sigma_{0}}\right)-{\left(\frac{\varrho \;a}{2\;\sigma_{0}}\right)}^{2}\left(\frac{\frac{\varrho \;a}{2\;\sigma_{0}}}{2\;\sigma_{0}\;\mathrm{asinh}\left(\frac{\varrho \;a}{2\;\sigma_{0}}\right)}-\sqrt{1+{\left(\frac{\varrho \;a}{2\;\sigma_{0}}\right)}^{2}\;}\right)}, \end{array}$$

which, introducing the non-dimensional parameter $\bar{\xi }$,

(35) $$\bar{\xi }=\frac{\varrho \;a}{2\;\sigma_{0}},$$

can be written as

(36) $$E_{t,eq,0}=\frac{\;\sqrt{1+{\bar{\xi }}^{2}}\;E}{\frac{{\bar{\xi }}^{2}}{3}\;\frac{E}{\sigma_{0}}+\frac{1}{8}\;\sqrt{1+{\bar{\xi }}^{2}\;}\left[5+2\;{\bar{\xi }}^{2}\right]+\frac{3}{8\;\bar{\xi }}\;\;\mathrm{asinh}\bar{\xi }-{\bar{\xi }}^{2}\left(\frac{\bar{\xi }}{\;\mathrm{asinh}\bar{\xi }}-\sqrt{1+{\bar{\xi }}^{2}\;}\right)},$$

or, equivalently, stressing the dependency on the cable sag f

(37) $$\begin{array}{c} E_{t,eq,0}\\ =\frac{\sqrt{1+{\left(4\;\frac{f}{a}\right)}^{2}}\;E}{\frac{1}{3}{\left(4\;\frac{f}{a}\right)}^{2}\frac{E}{\sigma_{0}}+\frac{1}{8}\sqrt{1+{\left(4\;\frac{f}{a}\right)}^{2}\;}\left[5+2{\left(4\;\frac{f}{a}\right)}^{2}\right]+\frac{3}{8}\;\left(\frac{a}{4\;f}\right)\;\mathrm{asinh}\left(4\;\frac{f}{a}\right)-{\left(4\;\frac{f}{a}\right)}^{2}\left(\frac{4\;\frac{f}{a}}{\;\mathrm{asinh}\left(4\;\frac{f}{a}\right)}-\sqrt{1+{\left(4\;\frac{f}{a}\right)}^{2}\;}\right)}. \end{array}$$

### 2.3. The Non-Linear Constitutive Law for a Cable with Fixed Ends

In case both ends of the cable are fixed (Figure 4), i.e., the inextensible length of the cable is not varying, the virtual displacement dx entirely results in a variation of the chord length: da=dx. In this case, recalling Equation (16) and, again, Equations (4), (5) and (15) becomes

(38) $$N_{0}\;da+\overset{a}{\underset{0}{\int }}qdy_{el}\;dx+\overset{a}{\underset{0}{\int }}q\left(\frac{\partial y}{\partial N_{0}}dN_{0}+\frac{\partial y}{\partial a}da\right)dx=\overset{L}{\underset{0}{\int }}\frac{N}{A_{0}}\frac{dN}{E\;A_{0}}A_{0}\;ds,$$

from which one obtains the analogous of Equation (25)

(39) $$N_{0}\;\;da+\overset{a}{\underset{0}{\int }}q\frac{\partial y_{el}}{\partial N_{0}}dN_{0}\;\;dx+\overset{a}{\underset{0}{\int }}q\left(\frac{\partial y}{\partial N_{0}}dN_{0}+\frac{\partial y}{\partial a}da\right)dx=\overset{L}{\underset{0}{\int }}\frac{N_{0}}{\cos\theta E\;A_{0}}\;\frac{dN_{0}}{\cos\theta }\;ds.$$

Remembering the previously calculated contributions, Equations (23) and (28), and given that it is

(40) $$\overset{a}{\underset{0}{\int }}q\frac{\partial y}{\partial a}da\;dx={\left(\frac{q\;a}{2\;N_{0}}\right)}^{2}da,$$

it results:

(41) $$\begin{array}{cc} \left[1+{\left(\frac{q\;a}{2\;N_{0}}\right)}^{2}\right]\;da & \\ & =\frac{q^{2}a^{3}}{12\;N_{0}^{3}}\;dN_{0}\\ & +\frac{\;dN_{0}}{8\;E\;A_{0}}\;\{a\;\sqrt{1+{\left(\frac{q\;a}{2\;N_{0}}\right)}^{2}\;}\left[5+2{\left(\frac{q\;a}{2\;N_{0}}\right)}^{2}\right]+6\;\frac{N_{0}}{q}\;\mathrm{asinh}\left(\frac{q\;a}{2\;N_{0}}\right)\\ & -\frac{2\;q^{2}a^{3}}{N_{0}^{2}}\left(\frac{\frac{q\;a}{2\;N_{0}}}{\mathrm{asinh}\frac{q\;a}{2\;N_{0}}}-\sqrt{1+{\left(\frac{q\;a}{2\;N_{0}}\right)}^{2}\;}\right)\}. \end{array}$$

Dividing both members of Equation (41) by a, the variation of the chord deformation

(42) $$d\varepsilon =\frac{da}{a},$$

can be derived

(43) $$\begin{array}{cc} \left[1+{\left(\frac{\varrho \;a}{2\;\sigma_{0}}\right)}^{2}\right]\;d\varepsilon & =\frac{\varrho^{2}a^{2}}{12\;\sigma_{0}^{3}}\;d\sigma_{0}\\ & +\frac{\;d\sigma_{0}}{8\;E\;}\;\{\;\sqrt{1+{\left(\frac{\varrho \;a}{2\;\sigma_{0}}\right)}^{2}\;}\left[5+2{\left(\frac{\varrho \;a}{2\;\sigma_{0}}\right)}^{2}\right]+6\;\frac{\sigma_{0}}{\rho \;a}\;\mathrm{asinh}\left(\frac{\varrho \;a}{2\;\sigma_{0}}\right)\\ & -\frac{2\;\varrho^{2}a^{2}}{\sigma_{0}^{2}}\left(\frac{\frac{\varrho \;a}{2\;\sigma_{0}}}{\mathrm{asinh}\left(\frac{\varrho \;a}{2\;\sigma_{0}}\right)}-\sqrt{1+{\left(\frac{\varrho \;a}{2\;\sigma_{0}}\right)}^{2}\;}\right)\}, \end{array}$$

where ϱ is the specific load (Equation (33)). In this case, the tangent elastic modulus expressing the non-linear constitutive law of the equivalent tie-rod is

(44) $$\begin{array}{c} E_{t,eq,a}=\frac{d\sigma_{0}}{d\varepsilon }\\ =\frac{\;\left[1+{\left(\frac{\varrho \;a}{2\;\sigma_{0}}\right)}^{2}\right]\;E}{\frac{1}{3}{\left(\frac{\varrho \;a}{2\;\sigma_{0}}\right)}^{2}\frac{E}{\sigma_{0}}+\frac{1}{8}\;\sqrt{1+{\left(\frac{\varrho \;a}{2\;\sigma_{0}}\right)}^{2}\;}\left[5+2{\left(\frac{\varrho \;a}{2\;\sigma_{0}}\right)}^{2}\right]+\frac{3}{4}\;\frac{\sigma_{0}}{\rho \;a}\;\mathrm{asinh}\left(\frac{\varrho \;a}{2\;\sigma_{0}}\right)-{\left(\frac{\varrho \;a}{2\;\sigma_{0}}\right)}^{2}\left(\frac{\frac{\varrho \;a}{2\;\sigma_{0}}}{2\;\sigma_{0}\;\mathrm{asinh}\left(\frac{\varrho \;a}{2\;\sigma_{0}}\right)}-\sqrt{1+{\left(\frac{\varrho \;a}{2\;\sigma_{0}}\right)}^{2}\;}\right)}, \end{array}$$

or, equivalently,

(45) $$E_{t,eq,a}=\frac{\;\left(1+{\bar{\xi }}^{2}\right)\;E}{\frac{{\bar{\xi }}^{2}}{3}\;\frac{E}{\sigma_{0}}+\frac{1}{8}\;\sqrt{1+{\bar{\xi }}^{2}\;}\left[5+2\;{\bar{\xi }}^{2}\right]+\frac{3}{8\;\bar{\xi }}\;\;\mathrm{asinh}\bar{\xi }-{\bar{\xi }}^{2}\left(\frac{\bar{\xi }}{\;\mathrm{asinh}\bar{\xi }}-\sqrt{1+{\bar{\xi }}^{2}\;}\right)},$$

or, as a function of the cable sag f,

(46) $$\begin{array}{c} E_{t,eq,a}\\ =\frac{\left[1+{\left(4\;\frac{f}{a}\right)}^{2}\right]\;E}{\frac{1}{3}{\left(4\;\frac{f}{a}\right)}^{2}\frac{E}{\sigma_{0}}+\frac{1}{8}\sqrt[{}]{1+{\left(4\;\frac{f}{a}\right)}^{2}\;}\left[5+2{\left(4\;\frac{f}{a}\right)}^{2}\right]+\frac{3}{8}\;\left(\frac{a}{4\;f}\right)\;asinh\left(4\;\frac{f}{a}\right)-{\left(4\;\frac{f}{a}\right)}^{2}\left(\frac{4\;\frac{f}{a}}{\;asinh\left(4\;\frac{f}{a}\right)}-\sqrt[{}]{1+{\left(4\;\frac{f}{a}\right)}^{2}\;}\right)}. \end{array}$$

### 2.4. Preliminary Remarks

Looking at the expressions of the equivalent elastic moduli, Equations (34) and (44), or Equations (37) and (46), it can be remarked that their limits exactly match the expectations. In fact, considering that the equilibrium conditions imply that, for q≠0,

(47) $$\underset{\;\;\frac{f}{a}\;\to 0}{\lim}\;\sigma_{0}=\infty ,$$

and

(48) $$\underset{\;\;\;\frac{f}{a}\;\to \infty }{\lim}\sigma_{0}=0,$$

the limits of $E_{t,eq,0}$ and $E_{t,eq,a}$ are

(49) $$\underset{\;\;\frac{f}{a}\;\to 0}{\lim}\;E_{t,eq,0}=E;\underset{\;\;\frac{f}{a}\;\to 0}{\lim}\;E_{t,eq,a}=E,\;\mathrm{and}$$

(50) $$\underset{\;\;\;\frac{f}{a}\;\to \infty }{\lim}E_{t,eq,0}=0;\;\underset{\;\;\;\frac{f}{a}\;\to \infty }{\lim}E_{t,eq,a}=0.$$

In addition, on a given set of conditions, the rigidity of the cable with fixed ends is higher than the rigidity of the cable with one end running on a fixed pulley. This aspect was already noticed for cables loaded by self-weight in [28,29].

It must be also underlined that the contribution to the equivalent axial stiffness due to the elastic increment of the cable ordinate, $dy_{el},$ is significant only for high values of the sag to chord ratio, f/a>0.1.

A particularly relevant feature of uniformly loaded cables is that the constitutive law of the equivalent tie-rod is a function of $\sigma_{0}/\varrho$, and $E/\sigma_{0}$: as the upper limit of $\sigma_{0}$ is controlled by the tensile strength, $f_{t}$, of the cable material, and $\sigma_{0}$ depends on ϱ via Equation (5), the performances of the cable thus pivot on the apparent specific strength, $f_{t}/\bar{\rho }$, and on the apparent specific stiffness, $E/\bar{\rho }$, of the cable material, being $\bar{\rho }$

(51) $$\bar{\rho }=\frac{\varrho }{g},\;$$

a sort of equivalent density, obtained regarding the specific load as an equivalent specific weight. Expressed in this way, governing parameters are analogous to the specific strength, $f_{t}/\rho$, and to the specific stiffness, E/ρ, respectively, characterizing the behavior of a cable, made with a material whose density is ρ, transversally loaded by the self-weight only, as extensively discussed in [28]. Anyhow, it must be stressed that a substantial difference exists between the two cases: in fact, while density ρ is a material property, the equivalent density $\bar{\rho }$ is not; consequently, cables made by different materials can exhibit similar behaviors, provided their apparent specific strength, $f_{t}/\bar{\rho }$, and their apparent specific stiffness, $E/\bar{\rho }$, are the same.

The expressions are valid also for creep sensitive materials, for which long term phenomena are relevant, by replacing the Young modulus of the material with the appropriate age-adjusted effective elastic modulus, taking into account the time dependency by means of a suitable creep coefficient [35].

## 3. Parametric Studies

The formulae previously derived allow to directly obtain the tangent elastic modulus of the equivalent tie-rod once the mechanical properties of the cable material, the cable geometry, and the applied load are known. However, in the author’s opinion, the most effective graphical representation of the ratio between the equivalent elastic modulus and the elastic modulus of the cable material is as a function of the sag to chord ratio, f/a, and of the normal stress to elastic modulus ratio, $\sigma_{0}/E$. For this reason, in the following, the constitutive laws of the equivalent tie-rod are plotted as a function of the previously cited relevant parameters.

From the operational point of view, the sag to chord ratio is commonly in the range 0–0.125, but in the following we explore the range 0–1.0, independently of its practical feasibility. Regarding the ratio $\sigma_{0}/E$, some additional consideration is necessary to fix its upper limit. Although currently adopted materials for structural cables are steel, aramid fiber, and carbon fiber [28,36], for the sake of the present study, we can explore a much more general set of natural and artificial materials, covering in principle every material suitable to build ropes and cables, as summarized in Table 1. In the Table, density, elastic modulus, ultimate strength, and ratios between the ultimate strength and the elastic modulus are given for relevant fibers. The mechanical properties of the cable materials reported in Table 1 have been derived summarizing data coming from several sources: the interested reader can specifically refer not only to the relevant literature [30,37], but also to some web sources [38,39], which provide links to cable material producers, too.

Looking at the Table, it clearly results that for most materials $f_{t}/E<1\%$; for polybenzoxazole (PBO), and carbon fibers is $f_{t}/E=1.5-2\%$; for aramid fibers, and liquid crystal aromatic polyester (LCP) fibers $f_{t}/E=2.5-4.5\%$; while nylon and polyester fibers exhibit higher values. Moreover, copper and aluminum, which are very important in terms of applications, are characterized by very small values of $f_{t}/E,$ being $f_{t}/E=0.15\%$, and $f_{t}/E=0.22\%$, respectively. As the design limit of $\sigma_{0}$ is a fraction of the ultimate strength of the material, in the following, we consider that $\sigma_{0}/E$ varies in the interval $\left[0,\;0.02\right]$.

### 3.1. The Equivalent Elastic Modulus of Cables Running on Fixed Pulleys

**Figure 5:**
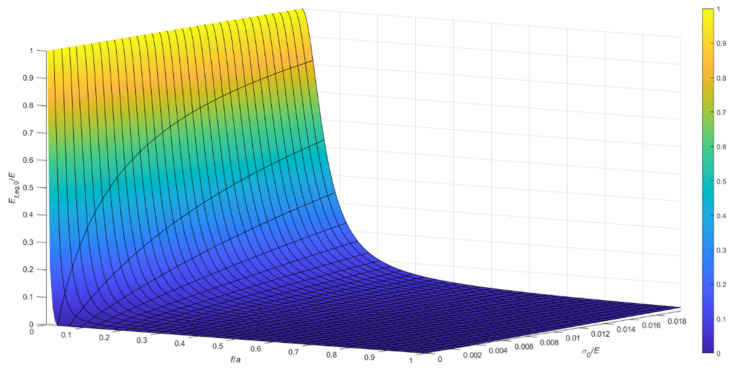
$E_{t,eq,0}/E-f/a-\sigma_{0}/E$ diagram for a cable running on a fixed pulley: da=0.

**Figure 6:**
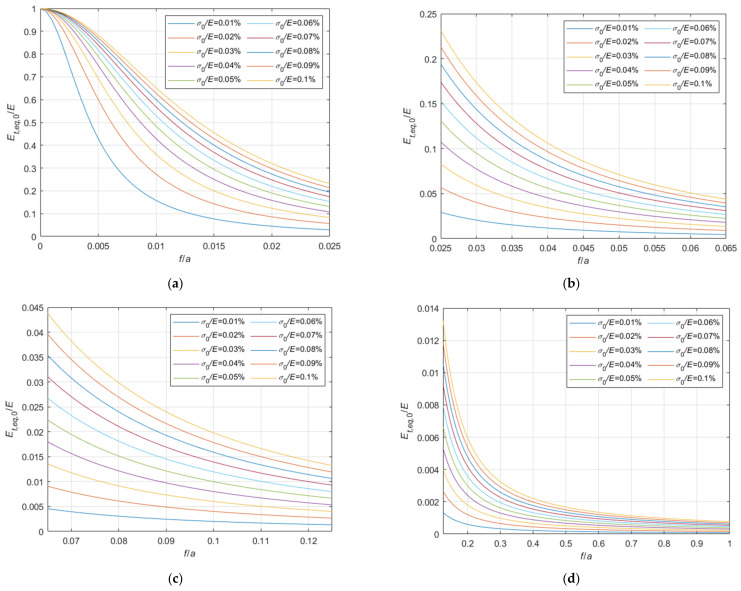
$E_{t,eq,0}/E-f/a$ diagrams parameterized in terms of $\sigma_{0}/E$ ($\sigma_{0}/E\in \left[0.01\%,\;0.1\%\right]):$ (**a**) 0<f/a ≤ 0.03; (**b**) 0.03<f/a ≤ 0.08; (**c**) 0.08<f/a ≤ 0.2; (**d**) 0.2<f/a ≤ 1.0.

**Figure 7:**
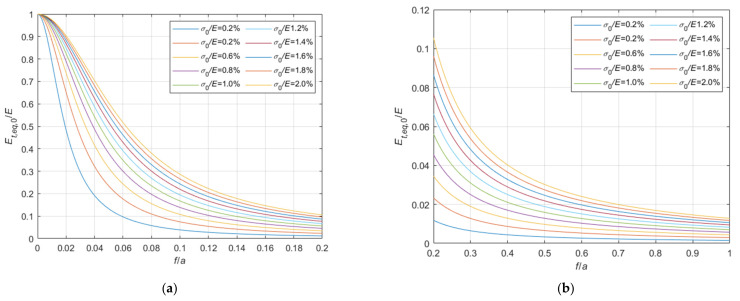
$E_{t,eq,0}/E-f/a$ diagrams parameterized in terms of $\sigma_{0}/E$ ($\sigma_{0}/E\in \left[0.2\%,\;2.0\%\right]):\;$(**a**) 0<f/a ≤ 0.2; (**b**) 0.2<f/a ≤ 1.0.

**Figure 8:**
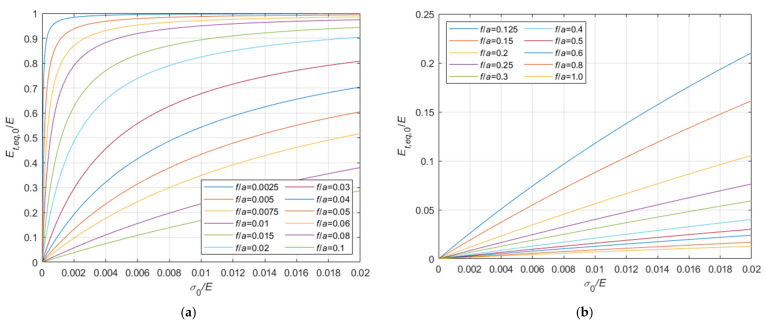
$E_{t,eq,0}/E-\sigma_{0}/E$ diagrams parameterized in terms of f/a: (**a**) 0.0025 ≤ f/a ≤ 0.1; (**b**) 0.125 ≤ f/a ≤ 1.0.

Considering the case of cable running on a fixed pulley, the ratio, $E_{t,eq,0}/E$, between the tangent elastic modulus of the equivalent tie-rod, $E_{t,eq,0}$, and the elastic modulus of the cable material, derived from Equations (34), (36), and (37), is diagrammatically represented in the 3D-graph of Figure 5. To facilitate the examination of the diagram, it is plotted in terms of contour lines in Figure 6, Figure 7 and Figure 8. More precisely, the $E_{t,eq,0}/E-f/a$ diagrams in Figure 6 and Figure 7 illustrate the contour lines obtained for $\sigma_{0}/E$ values belonging to the intervals $\left[0.01\%,\;0.1\%\right]$, and $\left[0.2\%,\;2.0\%\right]$, respectively, while the $E_{t,eq,0}/E-\sigma_{0}/E$ diagrams in Figure 8a,b show the contour lines for sag to chord ratios, f/a, varying in the range $\left[0.0025,\;0.1\right]$, and $\left[0.125,\;1.0\right]$, respectively.

### 3.2. The Equivalent Elastic Modulus of Cables with Fixed Ends

**Figure 9:**
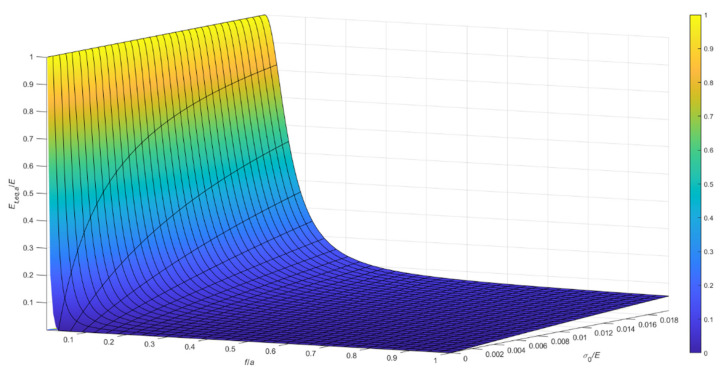
$E_{t,eq,0}/E-f/a-\sigma_{0}/E$ diagram for a cable with fixed ends.

Adopting criteria similar to those illustrated in Section 3.1, the tangent elastic modulus of the equivalent tie-rod, $E_{t,eq,a}$, can be calculated also in case the ends of the cable are fixed. The ratio, $E_{t,eq,a}/E$, is represented in the 3D-graph of Figure 9.

**Figure 10:**
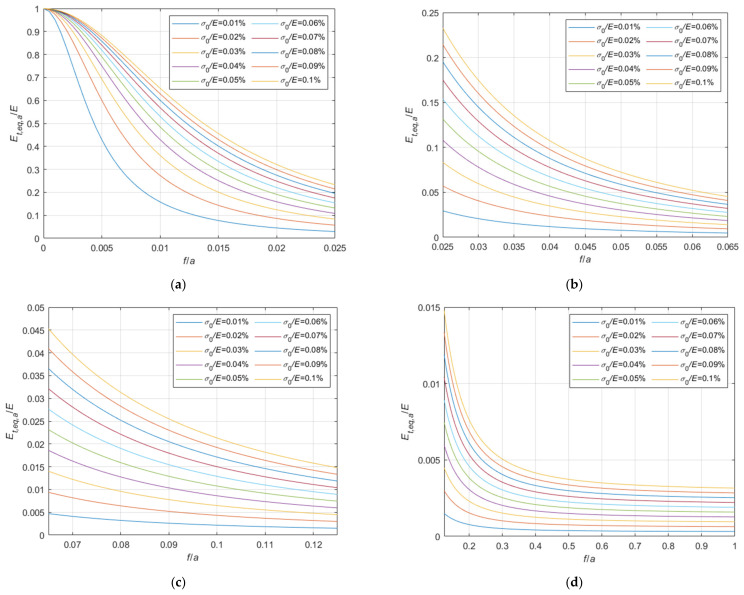
$E_{t,eq,0}/E-f/a$ diagrams parameterized in terms of $\sigma_{0}/E$ ($\sigma_{0}/E\in \left[0.01\%,\;0.1\%\right]):\;$(**a**) 0<f/a ≤ 0.025; (**b**) 0.025<f/a ≤ 0.05; (**c**) 0.05<f/a ≤ 0.1; (**d**) 0.1<f/a ≤ 1.0.

**Figure 11:**
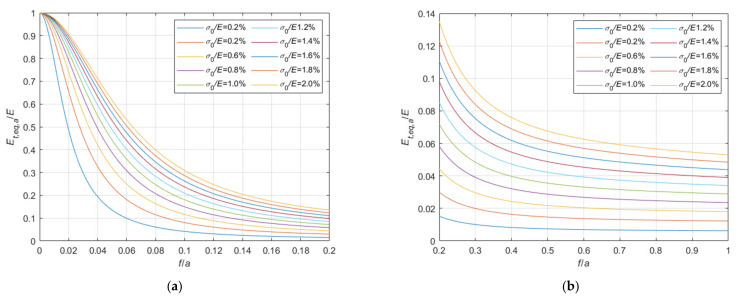
$E_{t,eq,a}/E-f/a$ diagrams parameterized in terms of $\sigma_{0}/E$ ($\sigma_{0}/E\in \left[0.2\%,\;2.0\%\right]):\;$(**a**) 0<f/a ≤ 0.2; (**b**) 0.2<f/a ≤ 1.0.

**Figure 12:**
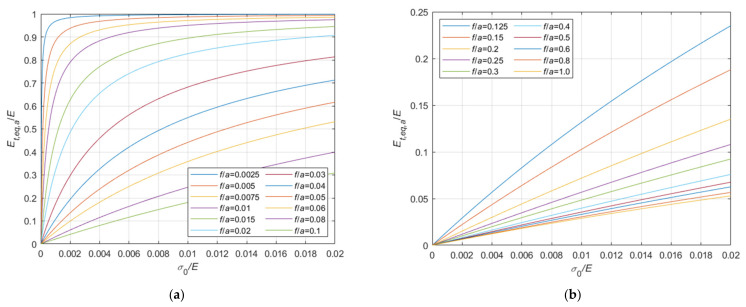
$E_{t,eq,a}/E-\sigma_{0}/E$ diagrams parameterized in terms of f/a: (**a**) 0.0025 ≤ f/a ≤ 0.1; (**b**) 0.125 ≤ f/a ≤ 1.0.

Again, to facilitate the examination, the diagram is plotted in terms of contour lines in Figure 10, Figure 11 and Figure 12. The $E_{t,eq,a}/E-f/a$ diagrams in Figure 10 and Figure 11 illustrate the contour lines obtained for $\sigma_{0}/E$ values belonging to the intervals $\left[0.01\%,\;0.1\%\right]$, and $\left[0.2\%,\;2.0\%\right]$, respectively, while the $E_{t,eq,a}/E-\sigma_{0}/E$ diagrams in Figure 12a,b show the contour lines for sag to chord ratios, f/a, varying in the range $\left[0.0025,\;0.1\right]$, and $\left[0.125,\;1.0\right]$, respectively.

## 4. Discussion

**Figure 13:**
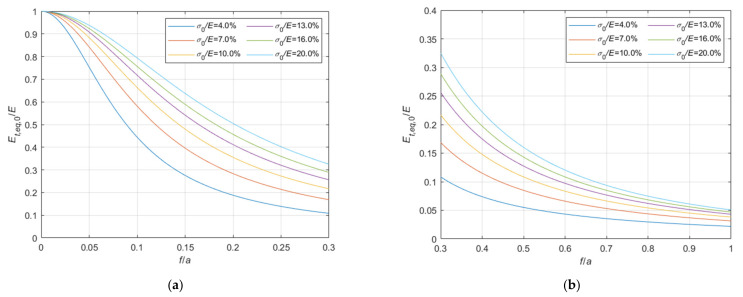
$E_{t,eq,0}/E-f/a$ diagrams parameterized in terms of $\sigma_{0}/E$ ($\sigma_{0}/E\in \left[4.0\%,\;20.0\%\right]):\;$(**a**) 0<f/a ≤ 0.3; (**b**) 0.3<f/a ≤ 1.0.

**Figure 14:**
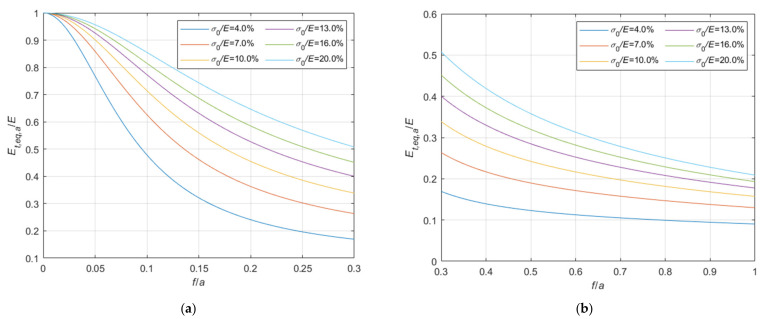
$E_{t,eq,a}/E-f/a$ diagrams parameterized in terms of $\sigma_{0}/E$ ($\sigma_{0}/E\in \left[4.0\%,\;20.0\%\right]):\;$(**a**) 0<f/a ≤ 0.3; (**b**) 0.3<f/a ≤ 1.0.

The examination of the diagrams demonstrates that, as expected, the ratio between the tangent modulus of the equivalent tie-rod and the elastic modulus of the material, $E_{t,eq}/E$:

- decreases as the sag to chord ratio, f/a, increases and that the reduction rate is particularly marked in the region where f/a and $\sigma_{0}/E$, are low;
- it is a quasi-linear function of the stress when f/a≥0.08;
- rises as the load, and then the ratio $\sigma_{0}/E$, increases; again, the increment rate is particularly marked in the region where f/a and $\sigma_{0}/E$, are low;
- for this reason, for creep sensitive materials, when the age-adjusted moduli $E_{t,eq}\left(t\right)$, and $E\left(t\right)$ are taken into account, it results:
  (52) $$\frac{E_{t,eq}\left(t\right)}{E\;\left(t\right)}>\frac{E_{t,eq}}{E},$$
  since the effect of the creep is an increase of the ratio $\sigma_{0}/E\left(t\right)$, as better illustrated in the diagrams of Figure 13 and Figure 14, where the $E_{t,eq,0}/E-f/a$ diagrams, and the $E_{t,eq,a}/E-f/a$ diagrams, respectively, are parameterized for high value of $\sigma_{0}/E$, in the interval $\left[4.0\%,\;20\%\right]$. Moreover, these diagrams, in combination with the ones previously given in Section 3, allow to consider all possible practical cases, including consideration of long terms effects.

It must be stressed that the influence of the boundary conditions, or, in other words, of the the end restraints, is relevant only for f/a≥0.08, and becomes very significant for flabby cables: in fact, the ratio $E_{t,eq,a}/E_{t,eq,0}$ is:

(53) $$\frac{E_{t,eq,a}}{E_{t,eq,0}}=\sqrt{1+{\left(4\frac{f}{a}\right)}^{2}}.$$

Considering how they have been derived, the proposed expressions allow one to assess the constitutive laws of the equivalent tie-rod for uniformly loaded cables, whatever the stress and the sag. Recalling that, said γ the specific weight of the cable material and ξ,

(54) $$\xi =\frac{\gamma \;a}{2\;\sigma_{0}},$$

the non-dimensional parameter of the catenary, analogous to $\bar{\xi }$ (see Equation (29)), the constitutive laws for the equivalent tie-rod of horizontal cables loaded by the self-weight [28] are given by:

(55) $$\frac{E_{t,eq,0}}{E}=\frac{\cosh\xi }{\frac{1}{12\;\xi }\left[\;9\;\sin h\xi +\sinh\left(3\;\xi \right)-24\sinh^{3}\xi +12\xi \;\sinh\xi \sinh\left(2\;\xi \right)\right]+\frac{E}{8\;\sigma_{0}}\left[\frac{\cos h\;\left(2\;\xi \right)}{2}-\frac{1}{\xi }\sinh\left(2\;\xi \right)\right]},$$

for the cable running on a fixed pulley at one end, and by

(56) $$\frac{E_{t,eq,a}}{E}=\frac{{\cos h}^{2}\xi }{\frac{1}{12\;\xi }\left[\;9\;\sin h\xi +\sinh\left(3\;\xi \right)-24\sinh^{3}\xi +12\xi \;\sinh\xi \sinh\left(2\;\xi \right)\right]+\frac{E}{8\;\sigma_{0}}\left[\frac{\cos h\;\left(2\;\xi \right)}{2}-\frac{1}{\xi }\sinh\left(2\;\xi \right)\right]},$$

for the cable with fixed ends, it is thus possible to assess parabolic or catenary cables, whichever the sag to chord ratio and the stress level.

### 4.1. Comparison with “Historical” Formulae

To further enrich the discussion, in the present Subsection the results obtained with the proposed formulae are compared with those obtained using the most used “historical” formulae, namely Dischinger’s formula [1,2], Equation (1), and Irvine’s formula [10], Equation (7), deriving the horizontal component of the tensile force on the cable from Equation (5), and assuming q≅p. Regarding Irvine’s formula, they are considered both expressions of the virtual length of the cable, $L_{e}$: the simplified version, Equation (11), and the accurate version, Equation (10). In the following, for the sake of completeness, also the case of cable with one end running on a fixed pulley is considered, even if it is not contemplated by the traditional formulae.

Although these historical expressions are normally used also for catenary cables, we will discuss here only uniformly loaded cables. Further information about catenary cables is given in [28,29]; moreover, the assessment of the equivalent uniformly distributed load and of the equivalent parabolic shape of sagging cables transversally loaded by the self-weight only is not an easy task, involving subtle equilibrium considerations.

#### 4.1.1. Comparison with the Dischinger’s Formula Results

**Figure 15:**
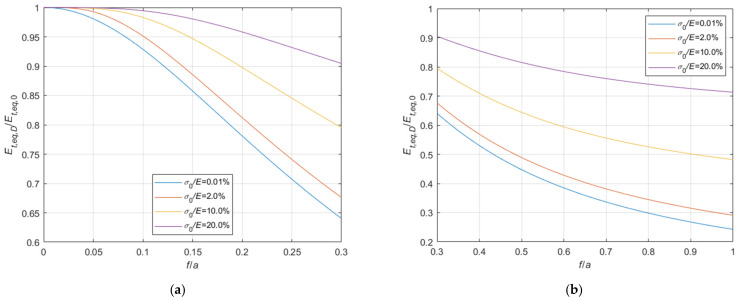
$E_{t,eq,D}/E_{t,eq,0}-f/a$ diagrams parameterized in terms of $\sigma_{0}/E$ ($\sigma_{0}/E\in \left\{0.01\%;2.0\%;10.0\%;20.0\%\right\}):\;$(**a**) 0<f/a ≤ 0.3; (**b**) 0.3<f/a ≤ 1.0.

**Figure 16:**
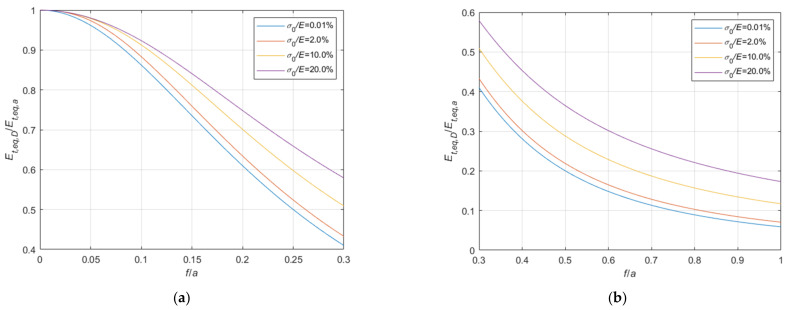
$E_{t,eq,D}/E_{t,eq,a}-f/a$ diagrams parameterized in terms of $\sigma_{0}/E$ ($\sigma_{0}/E\in \left\{0.01\%;2.0\%;10.0\%;20.0\%\right\}):\;$(**a**) 0<f/a ≤ 0.3; (**b**) 0.3<f/a ≤ 1.0.

The ratios between the tangent elastic modulus of the equivalent tie-rod, evaluated by means of the Dischinger’s formula Equation (1), $E_{t,eq,D}$, and by using the expressions derived before, $E_{t,eq,0}$ (Equation (34)), and $E_{t,eq,a}$ (Equation (44)) are illustrated in Figure 15 and Figure 16, respectively, as a function of the sag to chord ratio f/a, parameterized with respect to four relevant values of the $\sigma_{0}/E$ ratio, namely, 0.1%, 2%, 10%, and 20%.

The diagrams clearly show that Dischinger’s formula provides acceptable results only when the sag to chord ratio is very small and the stress is relatively high. In any case, Dischinger’s formula systematically underestimates the correct value.

#### 4.1.2. Comparison with the Irvine’s Formula Results

**Figure 17:**
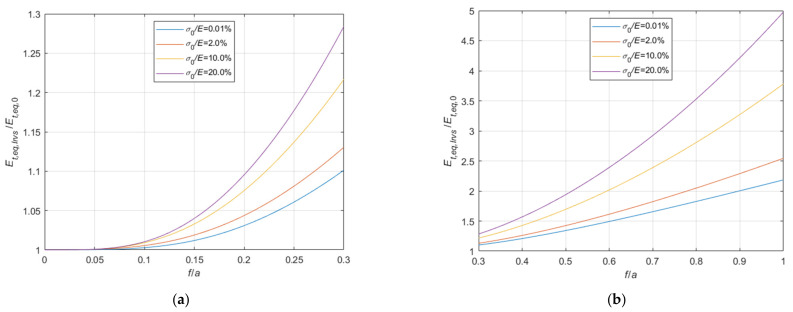
$E_{t,eq,Irvs}/E_{t,eq,0}-f/a$ diagrams parameterized in terms of $\sigma_{0}/E$ ($\sigma_{0}/E\in \left\{0.01\%;2.0\%;10.0\%;20.0\%\right\}):\;$(**a**) 0<f/a ≤ 0.3; (**b**) 0.3<f/a ≤ 1.0.

**Figure 18:**
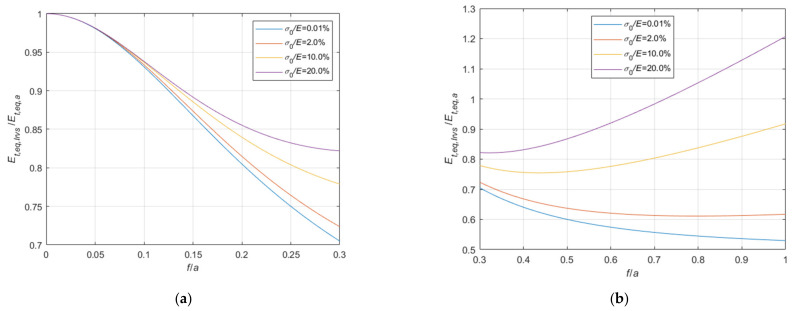
$E_{t,eq,Irvs}/E_{t,eq,a}-f/a$ diagrams parameterized in terms of $\sigma_{0}/E$ ($\sigma_{0}/E\in \left\{0.01\%;2.0\%;10.0\%;20.0\%\right\}):\;$(**a**) 0<f/a ≤ 0.3; (**b**) 0.3<f/a ≤ 1.

**Figure 19:**
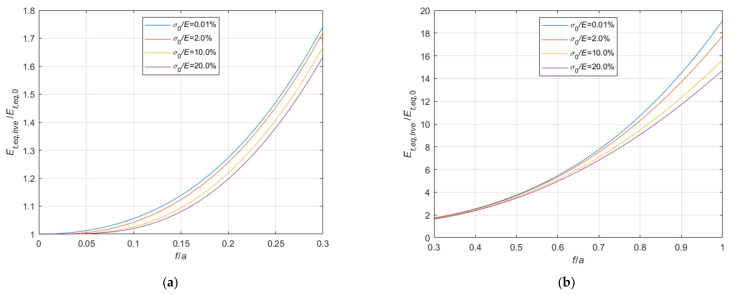
$E_{t,eq,Irve}/E_{t,eq,0}-f/a$ diagrams parameterized in terms of $\sigma_{0}/E$ ($\sigma_{0}/E\in \left\{0.01\%;2.0\%;10.0\%;20.0\%\right\}):\;$(**a**) 0<f/a ≤ 0.3; (**b**) 0.3<f/a ≤ 1.0.

**Figure 20:**
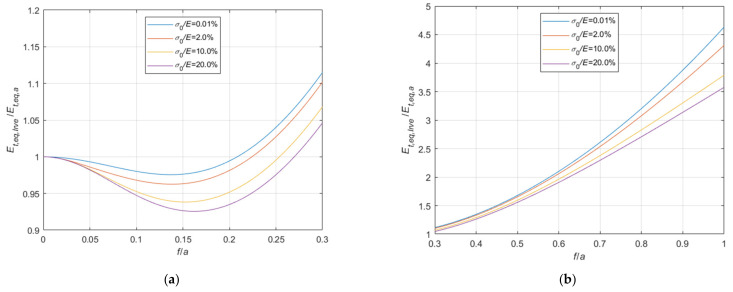
$E_{t,eq,Irve}/E_{t,eq,a}-f/a$ diagrams parameterized in terms of $\sigma_{0}/E$ ($\sigma_{0}/E\in \left\{0.01\%;2.0\%;10.0\%;20.0\%\right\}):\;$(**a**) 0<f/a ≤ 0.3; (**b**) 0.3<f/a ≤ 1.0.

The comparisons with Irvine’s formula results are summarized in Figure 17 and Figure 18, assuming $L_{e}$ expressed by the approximate Equation (11), and in Figure 19 and Figure 20, assuming $L_{e}$ expressed by the accurate Equation (10), analogously to what just done for Dischinger’s formula in Section 4.1.1. In the diagrams, $E_{t,eq,Irvs}$ indicates the equivalent tangent modulus obtained using simplified Equation (11), and $E_{t,eq,Irve}$ the equivalent tangent modulus obtained using Equation (10).

Analyzing the graphs, in the common case of cables with fixed ends,

(a) when the virtual length $L_{e}$ is calculated by means of the simplified expression, Equation (11), Irvine’s formula always underestimates the equivalent elastic modulus, leading to satisfactory results only for very small values of the sag to chord ratio f/a: in this field, it is more accurate than the Dischinger’s formula, being the ratio $E_{t,eq,Irvs}/E_{t,eq,a}$ practically insensitive to the stress level;
(b) when the virtual length $L_{e}$ is calculated by means of the accurate expression, Equation (10), Irvine’s formula can be adopted when the f/a is small. In its field of application, it generally leads to more precise evaluations of the equivalent modulus, both in comparison with Dischinger’s formula and with the simplified approach, anyhow underestimating the correct value, and resulting as much more sensitive to the stress level. In addition, as soon as f/a increases, the equivalent modulus is sensibly overestimated.

In the special case of a cable with the end running on a pulley, equivalent values calculated using the Irvine’s formula are always overestimated, especially when the accurate formula is adopted for $L_{e}$.

### 4.2. Comparison with Non-Linear FEA

Of course, for a sounder validation of the proposed approach, the analytical results should be analyzed in the light of relevant experimental studies results, especially concerning the context of the cables characterized by high values of the sag to chord ratio, where the proposed method provides estimates much more accurate than the traditional methods. Unfortunately, in the author’s knowledge, there are no available experimental results concerning the along the chord equivalent stiffness of such kind of cables. Nevertheless, a first validation of the method can be obtained comparing the theoretical results with those obtained with some refined non-linear finite element software. With this aim, cables characterized by different sag to chord ratios and by uniformly distributed loads of varying intensity have been studied using the Cosmos/M finite element software package.

To satisfactorily explore the range of the previously considered cases, four different values have been considered for the sag to span ratio: $f/a=\left\{0.04;0.1;0.25;1.0\right\}$ and six different values have been considered for the ratio between the horizontal component of the normal stress and the elastic modulus of the cable material: $\sigma_{0}/E=\left\{0.05\%;0.1\%;0.25\%;0.5\%;\;1.0\%;2.0\%\right\}.$

The cable has been discretized using 46 non-linear 2D truss elements, in such a way that the x-distance between two consecutive nodes was constant and equal to a/46.

**Figure 21:**
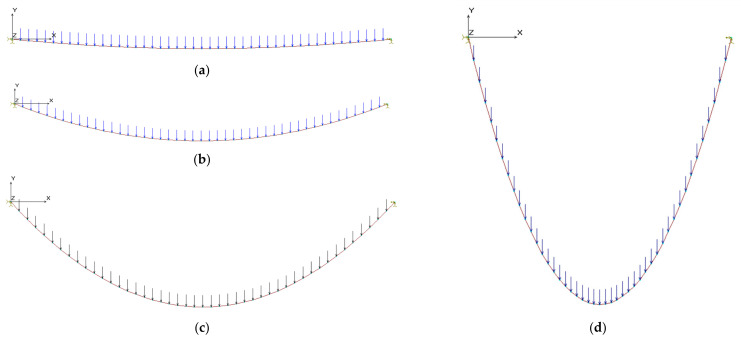
Finite element model of the cable: (**a**) f/a=0.04; (**b**) f/a=0.10; (**c**) f/a=0.25; (**d**) f/a=1.0.

During each non-linear run, the finite element model was first loaded by the uniformly distributed loads, represented by vertical forces applied on each node; subsequently, an imposed horizontal displacement of the 0.5% a was imposed to the right end of the cable. The FE models are summarized in Figure 21.

**Figure 22:**
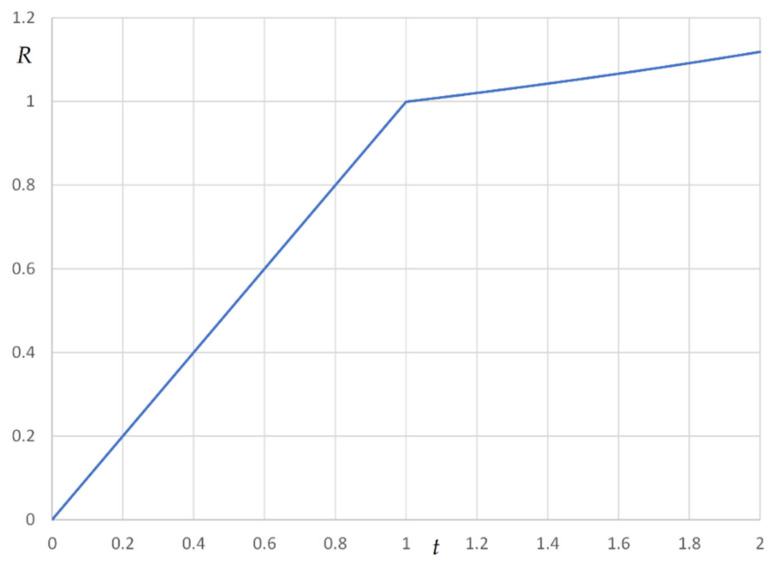
R−t diagram for the cable: loading phase 0≤t≤1; imposed displacement phase 1<t≤2.

The loading phase and the imposed displacement phase were both subdivided in 500 steps of constant amplitude, adopting an iterative Newton–Raphson algorithm to seek the convergence. The slope of the horizontal reaction–displacement curves in the initial stage of the imposed displacement phase allowed to derive the apparent stiffness. A typical reaction–displacement curve for flabby cables is illustrated in Figure 22, where the loading phase correspond to the first fictitious unitary time interval on the abscissa, and the imposed displacement phase to the second unit time interval. In the diagram is quite evident the hardening behavior of the cable in the displacement phase (t≥1).

**Figure 23:**
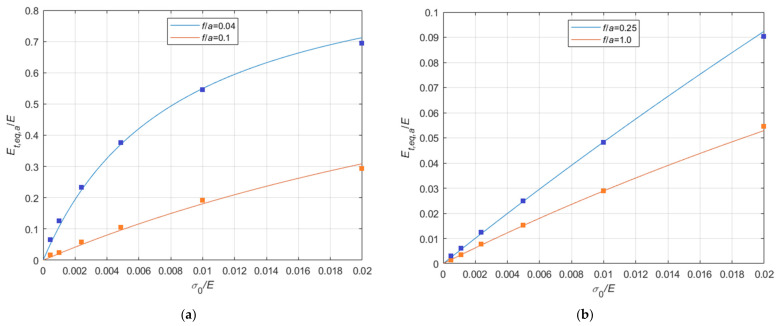
Comparison between $E_{t,eq,Irve}/E_{t,eq,a}-f/a$ theoretical curves parameterized in terms of $\sigma_{0}/E$ and nonlinear finite element analysis results ($\sigma_{0}/E\in \left\{0.01\%;2.0\%;10.0\%;20.0\%\right\}):\;$(**a**) 0<f/a ≤ 0.3; (**b**) 0.3<f/a ≤ 1.0.

The numerical results are compared with the pertinent theoretical curves in the diagrams in Figure 23a, referring to the cases f/a=0.04, and f/a=0.10, and in Figure 23b, referring to f/a=0.25, and f/a=1.0, where the numerical results are represented by shaded squares. The comparison seems satisfactory, even if some small discrepancy appears, especially for small values of the $\sigma_{0}/E$ ratios. In any case, further comparisons are necessary to fully validate the procedure.

The loading phase and the imposed displacement phase were both subdivided in 500 steps of constant amplitude, adopting an iterative Newton–Raphson algorithm to seek the convergence. The slope of the horizontal reaction–displacement curves in the initial stage of the imposed displacement phase allowed to derive the apparent stiffness. A typical reaction–displacement curve for flabby cables is illustrated in Figure 22, where the loading phase correspond to the first fictitious unit time interval on the abscissa, and the imposed displacement phase to the second unit time interval. In the diagram is quite evident that the hardening behavior of the cable in the displacement phase.

The numerical results are compared with the pertinent theoretical curves in the diagrams in Figure 23a referring to the cases f/a=0.04, and f/a=0.10, and in Figure 23b, referring to f/a=0.25, and f/a=1.0, where the numerical results are represented by shaded squares.

The comparison seems satisfactory, even if some small discrepancy appears, especially for small values of the $\sigma_{0}/E$ ratios. In any case, further comparisons are necessary to fully validate the procedure.

## 5. Conclusions

A traditional and very effective approach to assess the non-linear behavior of cable structures consists in replacing the cables with equivalent tie rods characterized by suitable non-linear constitutive laws, which have been the subject of several research works. Aiming to enlarge the field of application of the classical formulae, based on drastic simplifications, overcoming the severe limitations of the traditional approaches, analytical expressions of the non-linear constitutive laws of equivalent tie rod are proposed for horizontal cables transversally loaded by uniformly distributed loads, distinguishing two relevant cases, according as the length of the cable can vary or not:

- in the first case, rather new, an extremity of the cable is fixed, and the other end moves on a fixed pulley, so leaving unchanged the chord length; while
- in the second case, the one traditionally considered, both ends of the cable are fixed, so that the unstrained length of the cable is unaffected.

The expressions, derived by applying the general form of the theorem of virtual work, can be applied independently on the cable material, on the sag to chord ratio, on the load intensity, and on the stress level, so allowing the replacement of the whole cable with an appropriate single equivalent non-linear tie rod. Moreover, the expressions can be easily modified to include long-term effects, by introducing an appropriate age-adjusted elastic modulus, for example accounting for the creep coefficient.

Starting from a wide parametric study, exhaustive sets of curves are provided and commented for each relevant operational case, underlying the capabilities of the method, also in comparison with the traditional approaches. Finally, a first validation is obtained comparing the theoretical predictions with the numerical results obtained with a refined nonlinear finite element analysis.

## Figures and Tables

**Table 1:** Relevant mechanical properties of natural and artificial cable and rope materials.

Cable Material	Density [kg/m^3^]	E [GPa]	$f_{t}$ [MPa]	$\frac{f_{t}}{E}$
Aramid fiber (high modulus)	1440	112	3000	2.68%
Aramid fiber (normal modulus)	1440	70.5	2900	4.11%
Liquid crystal aromatic polyester (LCP) fiber	1410	66	2830	4.29%
Polybenzoxazole (PBO) fiber	1560	270	3950	1.46%
Carbon fiber 1	1560	170	2500	1.47%
Carbon fiber 2 ^1^	1800	240	4850	2.02%
Steel strand	7850	180	1770	0.98%
Steel wire	7850	206	1900	0.92%
Copper	8940	110	240	0.22%
Aluminum	2700	69	105	0.15%
Nylon fiber	1140	4.56	610	13.38%
Polyester fiber	1380	13.8	790	5.72%
Cotton rope	1540	7.9	225	2.85%
Hemp rope	1490	32	300	0.94%
Flax rope	1540	27	340	1.26%
Jute rope	1500	25.8	230	0.89%
Abaca (Manila hemp) rope	1320	30	300	1.00%
Sisal	1320	30	250	0.83%
Silk (silkworm)	1320	10	650	6.50%
Silk (spider)	1100	12	900	7.50%

^1^ Carbon fiber for structural applications [31].
